# Triggers of Agitation in Psychiatric Hospitalization Ward According to Professional Experience Questionnaire

**DOI:** 10.3390/ijerph19042014

**Published:** 2022-02-11

**Authors:** Irene Ortiz-Sandoval, María Dolores Martínez-Quiles, Jesús López-Pérez, Agustín Javier Simonelli-Muñoz

**Affiliations:** 1 Faculty of Nursing, Campus de los Jerónimos s/n., Catholic University of Murcia, 30107 Murcia, Spain; ireneosesm@gmail.com; 2 Psychiatric Hospitalization, University Morales Meseguer Hospital, 30008 Murcia, Spain; doloresquiles@gmail.com; 3 Department of Anesthesiology, University Arrixaca Hospital, 30120 Murcia, Spain; jesuslpmed@gmail.com; 4 Department of Nursing, Physiotherapy and Medicine, University of Almeria, 04120 Almeria, Spain

**Keywords:** nursing, agitation trigger, professional experience, psychiatric hospitalization

## Abstract

Aim: To create and analyze an instrument to assess the possible agitation triggers of hospitalized psychiatric patients. Background: No tools exist for identifying according to a professional’s experience. Methods: Descriptive and cross-sectional study. The questionnaire of possible triggers of agitation behaviors of patients hospitalized in psychiatric wards according to professional experience (TAPE) was designed and analyzed. Results: The questionnaire was provided to 156 mental health workers (76.9% women, average work experience: 10.15 ± 8 years, 46.8% were nurses specialized in mental health, and 21.2% psychiatrists). A good internal consistency was obtained, with a Cronbach α value of 0.791 in the initial test, and 0.892 in the retest. The factorial analysis found four factors: factor 1 “personnel”, factor 2 “routines”, factor 3 “norms–infrastructure”, and factor 4 “clinic”. Factor 1 obtained the highest value, with a mean of 4.16 ± 0.63, highlighting the item “lack of specialized personnel” (mean 4.38 ± 0.81). The specialized professionals provided higher scores to the items from the factors associated with the training of the personnel and routines (*p* = 0.017; *p* = 0.042). Conclusions: The TAPE questionnaire is useful for identifying the possible triggers that could lead to situations of agitation of hospitalized patients.

## 1. Introduction

During psychomotor agitation, an increase in the motor activity is produced, provoked by organic or psychiatric causes; also, some drug-induced triggers of agitation may depend on pharmacogenomics [1]. These can rapidly escalate to aggressive patient behaviors, such as verbal aggressions or threats, damage to the property, themselves, other patients, or staff [2]. Psychomotor agitation is considered an acute emergency which requires identifying or discarding an organic cause and an immediate intervention to control the symptoms, to decrease the risk of lesions of the patient or other individuals [3].

The Project BETA guidelines were published by the American Association for Emergency Psychiatry [4] and provide detailed guidance on various aspects of patient management at risk or during psychomotor agitation. The guidelines for the management of psychomotor agitation include verbal de-escalation and pharmacological restraint. Drugs used in chemical restraint included olanzapine, haloperidol, droperidol, risperidol, flunitrazepam, midazolam, promethazine, ziprasidone, sodium valproate, or lorazepam. There is no clear superiority of any chemical method of managing psychomotor agitation [5]. When these above measures are not effective, physical restraint and/or isolation are required.

At the international level, work has been performed to reduce situations of agitation which can result in coercion measures (forced medication, mechanical restraint, or restriction), such as the EUNOMIA program in Europe [6] and the BETA project in the United States [7], due to the medical–legal aspects [8], post-traumatic stress of the patients, possible physical damage to the patients and professionals [4], and the high medical costs [9].

The UN 2020 report recognizes the need to protect, promote, and respect all human rights in the global response to mental health issues, and stresses that mental health and community services must incorporate a human rights perspective, not to cause any harm to the people who make use of them and to respect their dignity, their integrity, their enjoyment of legal capacity on an equal basis with others, their choices, and their inclusion in the community [10].

Different programs have been implemented at the international level to reduce agitation situations. For example, Bowers et al., through his program “Safewards” [11], proposed diverse actions on the staff, the physical environment, the patient’s community, the patient’s characteristics, and the regulatory framework. Huckshorn et al. presented the “six core strategies” model: leadership toward organizational change, use of data to inform practice, workforce development, use of seclusion/restraint prevention tools, consumer roles in inpatient settings, and debriefing techniques [12].

### Related Studies

Many international psychiatric studies have stressed the importance of better management of agitated patients, to avoid losing control and restrictive or isolation measures [2,9,11,13], with the prediction of risk of violence being a key component in clinical practice [14,15]. Novel technologies in monitoring such as emotion-aware computing propose improved algorithms for intelligent emotion-aware systems capable of predicting risk [16].

The agitation situations and the use of coercive measures can be reduced by learning verbal de-escalation techniques and the evaluation of risks [14].

In recent literature, many experts consider verbal de-escalation and environmental modification techniques as the main options for managing the aggressive patient, considering restraint as a last-resource strategy [17].

Risk assessment scales are commonly used to identify the risk of aggression [18]. These instruments help health personnel to identify and manage the risk of aggression in populations of hospitalized psychiatric patients to prioritize preventive interventions. The most utilized are The Overt Aggression Scale, OAS [19], Staff observation aggression scale, SOAS [20], Staff observation aggression scale-revised, SOAS-R [21], The Brøset Violence Checklist, BVC [22], Positive and Negative Syndrome Scale, PANSS [23], Excited Component of the Positive and Negative Syndrome Scale, PANSS-EC [24], and The Dynamic Appraisal of Situational Aggression, DASA [25].

It is important to also consider that external factors such as the organization of the institution, and personal situations and relationships, can also have an effect on the patient, and these can be modified. Agitation can also be incited by a combination of factors including patient, staff, and ward factors. [26,27,28,29,30].

Given the above, and due to the importance of the assessment of external risks, it is necessary to design a specific and reliable tool to assess the possible triggers that can lead to agitation situations in a hospital psychiatric ward. Therefore, the objective of the study was to create and validate a tool that could help with the identification of possible agitation triggers of hospitalized psychiatric patients, based on the professional experience of different psychiatric ward personnel.

The present manuscript is organized in the following manner: an introduction which describes the current situation of mechanical restraint of psychiatric patients, and the object of study; next, the material and methods section explains the type of study, the creation of the questionnaire and its items, and the factorial analysis; posteriorly, the results are described, which are compared and contrasted with past studies in the discussion section. Lastly, the research conclusions are presented, which detail the limitations and provide suggestions for further studies.

## 2. Materials and Methods

This is a descriptive and cross-sectional study that includes mental health personnel in Murcia (Spain). It took place from the end of December 2020 to the end of January 2021. Different types of psychiatric ward professionals participated in the study: psychiatrists, psychologists, mental health nurses, clinical aides, ward staff, psychiatric, mental health nurses, and psychology residents, who had previously provided services in mental health.

The study protocol was approved by the Ethics and Research Board at the HMM and complies with the Helsinki Declaration from 1964. The participants provided their consent when completing the questionnaire.

A total of 156 individuals, who had worked in mental health in the Region of Murcia (Spain), were included. The mean number of years worked was 10.1± 8 years. Most of the workers were women (76.9%, 120). All of them were mental health professionals and most worked in hospital psychiatric wards (38.5%) and mental health centers (34%). Additionally, 16.7% worked in other mental health resources, with the remaining 10.9% not working at the moment in mental health. As for their professional category, most of them were nurses specialized in mental health (46.8%), followed by psychiatrists (21.2%). The residents in training and nurse assistants totaled 13.4% and 9.6%, respectively. Lastly, the rest of the professionals were psychologists (5.1%) and ward staff (3.8%).

Personal and employment variables were studied, such as sex, professional category, years worked in mental health, and the place where they presently worked. The questionnaire was created by the research team with Google Forms and sent to the mental health personnel in the Region of Murcia (Spain), which includes 473 workers, according to the transparency portal of the Murcia Health Services [31]. The participants were previously informed about the characteristics of the study, as well as the final use of the data recorded.

### 2.1. The Questionnaire of Possible Triggers of Agitation Behaviors of Patients Hospitalized in Psychiatric Wards According to Professional Experience (TAPE)

The TAPE questionnaire was designed by the research team, comprised by two mental health nurses (one with experience in hospital psychiatric wards and mental health centers, and the other with experience in mental health research), and a psychiatrist from the hospital psychiatric ward. The generation of items was performed through a literature search of agitation triggers [2,3,4,13,14,18,19,20,21,24,26] and the professional experience of the research team [32]. The aim of the questionnaire was to uncover, according to the experience of mental health personnel, the possible triggers that can lead to agitation behaviors in hospital psychiatric wards. The Cronbach’s alpha coefficient for the subscales oscillated between 0.840 and 0.847. This questionnaire included 18 items scored with a 5-point Likert-type scale (1 = without risk of showing agitation behavior to 5 = greater risk of showing agitation behavior). The greater the score, the greater the worker’s perception that a trigger of agitation behavior could appear.

### 2.2. Statistical Analysis

The categorical variables are shown as absolute frequencies (percentages), while the continuous variables are shown as the mean ± SD (standard deviation). The Kolmogorov–Smirnov test was utilized to verify the normal distribution of the continuous data.

The statistical test utilized to analyze the reliability of the scale was the test–retest, with the calculation of the intraclass correlation coefficient (ICC) to measure the agreement between the quantitative measures obtained in the questionnaire. To analyze the internal consistency, Cronbach’s *α* was utilized, with a value of 0.700 being the minimum value desired.

An exploratory factor analysis (EFA) was performed to analyze the structure, the relationship between variables. EFA is a statistical technique that allows us to explore the underlying dimensions, constructs, or latent variables of the variables observed in a more precise manner. A principal component analysis (PCA) with varimax rotation was performed to determine the factorial loads. Before this analysis, the Kaiser–Meyer–Olkin (KMO) and Bartlett’s sphericity tests were performed to analyze the adequacy of the data for the EFA [33]. Only the factors with values higher than 1 were extracted, as these explained the greater degree of total variability, utilizing the criteria that the extracted components comprised at least 60% of the variance explained by the correlation matrix. For the factorial loads to be consistent, for an item to be part of the factor extracted, its value had to be the equal to or greater than 0.40.

For the bivariate analysis, Person’s correlation coefficient, Student’s t-test, and a one-way ANOVA were utilized.

A value of *p* < 0.05 was considered significant. The statistical analyses were performed with the SPSS v21 software for Windows (SPSS, Inc., Chicago, IL, USA).

## 3. Results

To analyze the reliability, a pilot study was conducted with a sample of 36 professionals, through the use of the test–retest technique (Table 1), by repeating the questionnaire 14–21 days after completing it for the first time. The Spearman–Brown’s coefficient was calculated, with a value of 0.862 obtained, indicating the good reliability of the questionnaire. In the analysis of the two tests, Cronbach’s alpha value, which measures the internal consistency of the items that comprise the questionnaire in the pilot test, were also obtained, with a value of 0.791 for the original test and a value of 0.892 for the retest (Table 1).

To facilitate the management and interpretation of the results, a factorial analysis of the TAPE questionnaire was performed. First, we determined if the criteria necessary for its application were met, by verifying the existence of an underlying structure shaped by four factors, in agreement with the Kaiser rules. As a group, they explained 60.6% of the variance. The factorial load of each item was satisfactory for their inclusion in the model, as the values were >0.40. After the rotation, factor 1 included five items related with “personnel”, factor 2 included six items related with “routines”, factor 3 included five items related with “norms–infrastructure”, and factor 4 included three items related to “clinic” (Table 2).

All the factors have Cronbach’s α values above 0.700. Factor 1 “personnel”, has a Cronbach’s α of 0.783; factor 2 “routines”, a Cronbach’s α of 0.752; factor 3 “norms–infrastructure”, a Cronbach’s α of 0.725, and factor 4 “clinic”, a Cronbach’s α of 0.706.

The average score of all the items was 3.49 ± 0.51. Factor 1 obtained the highest score, with a mean of 4.16 ± 0.63, highlighting the item “lack of specialized personnel”, with a mean of 4.38 ± 0.81 (Table 3).

Table 4 shows the association between the questionnaire of agitation triggers (TAPE) and the characteristics, both personal and work-related, of the professionals who worked in mental health. We can observe the lack of association of the scores with respect to the variable sex, in all of the factors and the questionnaire as a whole.

When the professional category was analyzed, despite the lack of differentiation with the values of the questionnaire when all the questions were considered, we found a significant difference through an ANOVA (*p* = 0.006) in the factorial analysis between the professional category and factor 1, related with the professional training of the personnel. After the posterior application of the post-hoc multiple comparison Bonferroni test of factor 1, we found significant differences (*p* < 0.041 and *p* < 0.007) between the professional groups ward staff and mental health and residents-in-training, respectively. The specialized professionals and the residents in-training in the same professional categories granted a greater importance to the items in factor 1 (mental health nurse 4.22 ± 0.57; psychiatrist 4.13 ± 0.67; psychologist 4.10 ± 0.59; and residents 4.44 ± 0.35). This difference was re-enforced in workers from mental health centers (4.32 ± 0.54 *p* = 0.013) (Table 4).

As for the area of mental health of these professionals, statistically significant differences were observed in the analysis of factor 1 (*p* = 0.013) associated with the training of the staff, with the lowest mean found in the Hospital Psychiatric Ward (3.96 ± 0.75) (Table 4).

When analyzing the association between the TAPE and the work experience measured in years, a negative and weak correlation was found with the total questionnaire (r = −0.282 *p* = 0.001) and factors 1, 2, and 3 (r = −0.18 *p* = 0.024; r = −0.31 *p* = 0.001; r = −0.19 *p* = 0.015), respectively, which indicated a slight tendency of assigning lower scores to the items from these factors, as the professional’s experience increased (Table 4).

## 4. Discussion

The objective of the present study was to design a simple tool to identify the possible agitation triggers in a hospital psychiatric ward according to the ward professionals’ experience. The TAPE questionnaire included 18 items and was validated by professionals who had worked in mental health, with the tool showing good internal consistency.

A review of the literature demonstrated the scarce number of studies conducted at hospital psychiatric wards and the difficulty in the assessment of the quality of the programs, services, and results of medical care [17], with differences found in the use of guides among centers and where even acute stress, such as a transplant, could be a trigger [34]. Future research should focus on the use of programs and homogenous guides such as the WHO Quality Rights Toolkit [35] between countries to reduce mechanical restraint [6]. We found that, specifically, the Assessment toolkit overview document can be used to assess and improve quality and human rights in mental health and social care facilities, through the evaluation tools, the implementation of improvements, and follow-up found within it [35].

Professional experience can help with the management of the agitated patient [31,36], and the decrease in the use of coercive measures, and this is where we find the importance of the present study.

Diverse research studies have shown the importance of modifying external factors, such as the organization of the institution, the specialization of the staff, and routines, given their influence on the state and evolution of the patient [2,11]. In our study, the professionals specialized in mental health, psychiatrists, psychologists, nurses, and residents in training, granted more importance to training in factor 1. This factor included involuntary admission, the lack of information about the patient, lack of communication with the team, lack of specialized staff/specialist, lack of training, and verbal de-escalation.

According to the TAPE questionnaire, more importance was given to the lack of specialized training of the health professionals as a possible trigger of agitation. This lack of training could be corrected by the hospital management board with the creation of specific job postings to cover these posts with specialized personnel. A systematic review, which recommended training the staff, also underlined verbal de-escalation techniques and evaluation of risks, such as the prediction of patients at risk of violence, and the reduction of mechanical/physical restraints. This review ultimately recommended teaching and training of health professionals, especially mental health nurses [14]. Similarly, the BETA report indicated that all the clinical personnel from an emergency service or psychiatric emergency services, should receive at least annual training on verbal de-escalation techniques and the prevention and management of aggressive behavior [4]. The second item that was highly scored in the TAPE was involuntary admission, in which the use of coercive measures, such as forced medication, isolation, and restriction, were utilized as the treatment for agitated patients, although they were associated with a greater degree of lesions for the patients and the staff (psychical and psychological), which could also affect the doctor–patient relationship [4]. 

In the present study, more importance was given to training in mental health centers (MHC) than in hospital wards. This could be because in Murcia, weekly clinical sessions are conducted at the MHC, with the participation of members of the healthcare team within their work day, and at the hospital wards, the clinical sessions are performed weekly, but without the participation of the nurses, assistants, or ward staff, as the patients are under a 24 h watch.

In our study, the greater the work experience, the lower the agitation triggers scored in factors 1, 2, and 3, with respect to the workers from other centers within the mental health network. This could be due to skill development, the trust in the means used by the specialized hospital workers in situations of agitation in their work environment. According to De Benedictis [26], in general, the staff members with experience calm the patients more effectively that the less experienced staff, and utilize confinement and restrain less often.

## 5. Conclusions

This study demonstrated that the TAPE questionnaire is a simple and easy tool to use, showing a good reliability. This is a useful instrument for identifying possible triggers of agitation at a hospital psychiatric ward, according to the experience of ward professionals. Further studies are needed to confirm if its use could allow us to intervene on these triggers and decrease the use of coercion methods.

### 5.1. Limitations

The TAPE was created and internally content validated by staff who worked in the Region of Murcia (Spain). To obtain an optimal content validity, experts in their respective fields should assess this questionnaire. More prospective studies are needed in other regions in Spain and at the international level, to externally validate this questionnaire for its use in clinical practice. The TAPE questionnaire will identify triggers for agitation. More studies are needed to see if intervention on these triggers produces a reduction in agitation situations.

There was no construct validation, no examination with related scales, and no testing of organizational variables. Additionally, the absence of prior hypotheses could alter the value of the association of the scale or subscales with the characteristics of the personnel.

Another potential limitation is to consider the mental health personnel as a group, because the findings may not be homogeneous.

### 5.2. Suggestions for Future Works

Lastly, more collaboration is needed between the managers from the different centers, to implement the questionnaire at their institutions along with the center’s staff, with the commitment to improve the trigger conditions. The authors suggest future intervention studies that improve the level of training of the personnel, and that assess their repercussion on the number and quality of the mechanical restraints.

## Figures and Tables

**Table 1:** Test–retest of the items in the pilot study.

		ICC (CI 95%)	F	*p*
1. Involuntary admission		0.604 (0.215–0.800)	2.525	0.004
2. Change from voluntary to involuntary		0.719 (0.444–0.858)	3.561	0.001
3. Schedules implemented		0.690 (0.387–0.844)	3.231	0.001
4. Bathroom supervision		0.765 (0.535–0.882)	4.26	0.001
5. Taking of medication		0.753 (0.511–0.875)	4.048	0.001
6. Smoker		0.799 (0.602–0.899)	4.979	0.001
7. Assigned diets		0.834 (0.671–0.916)	6.018	0.001
8. Water drinking monitoring		0.773 (0.550–0.885)	4.404	0.001
9. Lack of patient clinical information		0.795 (0.595–0.897)	4.886	0.001
10. TV use		0.836 (0.675–0.917)	6.101	0.001
11. Not allowing calls/visits		0.165 (−0.654–0.579)	1.198	0.041
12. Scarce infrastructures		0.681 (0.368–0.839)	3.132	0.001
13. Hospitalization unit closed		0.858 (0.719–0.928)	7.048	0.001
14. Not authorizing phone, laptop		0.728 (0.460–0.862)	3.671	0.001
15. Not authorizing clothes from home		0.854 (0.710–0.926)	6.835	0.001
16. Bad communication among care team		0.718 (0.442–0.858)	3.552	0.001
17. Lack of qualified/specialized personnel		0.482(−0.27–0.738)	1.93	0.03
18. Lack of personnel training courses		0.470 (−0.050–0.732)	1.886	0.034
Cronbach’s Alpha	test	0.791		
	retest	0.892		
Correlation between forms		0.758		
Spearman–Brown Coefficient		0.862		

ICC: intraclass correlation index.

**Table 2:** Factorial analysis of the questionnaire: matrix of rotated components.

Kaiser–Meyer–Olkin Test	0.804				
Bartlett’s Sphericity Test	<0.001				
Items		Factor 1 Personnel	Factor 2 Routines	Factor 3 Norms–Infrastructure	Factor 4 Clinic
1. Involuntary admission		0.482			
2. Change from voluntary to involuntary					0.478
3. Schedules implemented			0.746		
4. Bathroom supervision			0.787		
5. Medication schedule			0.594		
6. Smoker			0.410		
7. Diets					0.788
8. Water drinking monitoring					0.765
9. Lack of patient information		0.476			
10. TV use			0.535		
11. No phone/visits				0.718	
12. Scarce infrastructures				0.541	
13. Closed hospitalization unit				0.654	
14. Non phone/laptop use				0.669	
15. No house clothes/pajamas			0.475	0.522	
16. Bad team communication		0.702			
17. Lack of qualified/specialized personnel		0.893			
18. Lack of training/verbal de-escalation		0.850			
Self-values Variance		5.173 31.54%	2.341 14.27%	1.356 8.26%	1.083 6.60%

**Table 3:** Descriptive statistics of the TAPE.

	Min.	Max.	Mean	Std. dev.
Mean of factors and items	1	5	3.49	0.51
Factor 1 (1, 9, 16, 17, and 18)	1	5	4.16	0.63
Factor 2 (3, 4, 5, 6, 10, and 15)	1	5	2.89	0.67
Factor 3 (11, 12, 13, 14, and 15)	1	5	3.62	0.68
Factor 4 (2, 7, and 8)	1	5	3.14	0.76
1. Involuntary admission	1	5	4.31	0.74
2. Change from voluntary to involuntary	1	5	4.24	0.86
3. Schedules implemented	1	5	2.78	1.09
4. Bathroom supervision	1	5	2.48	0.96
5. Taking of medication	1	5	2.94	1.03
6. Smoker	1	5	3.92	0.97
7. Assigned diets	1	5	2.40	1.03
8. Water drinking monitoring	1	5	2.81	1.05
9. Lack of patient clinical information	1	5	3.84	0.93
10. TV use	1	5	2.46	0.91
11. Not allowing calls/visits	1	5	4.22	0.86
12. Scarce infrastructures	1	5	4.07	0.89
13. Hospitalization unit closed	1	5	3.57	1.10
14. Not authorizing phone, laptop	1	5	3.48	1.04
15. Not authorizing clothes from home	1	5	2.79	1.05
16. Bad communication among care team	1	5	4.06	0.91
17. Lack of qualified/specialized personnel	1	5	4.38	0.81
18. Lack of personnel training courses	1	5	4.25	0.92

**Table 4:** Association between the questionnaire on agitation triggers and the personal and employment factors of the personnel who work in MH.

Variables Investigated	Factor 1 Personnel	Factor 2 Routines	Factor 3 Norms–Infrastructure	Factor 4 Clinic	Total Questionnaire
Sex					
Male (*n* = 36)	4.03 ± 0.66	2.81 ± 0.71	3.48 ± 0.74	3.07 ± 0.88	3.39 ± 0.58
Female (*n* = 120)	4.20 ± 0.62	2.91 ± 0.65	3.67 ± 0.66	3.27 ± 0.73	3.53 ± 0.48
	*p* = 0.151	*p* = 0.442	*p* = 0.154	*p* = 0.503	*p* = 0.150
Professional Category					
Nurse mental health (*n* = 73)	4.22 ± 0.57	2.95 ± 0.60	3.64 ± 0.65	3.15 ± 0.75	3.54 ± 0.46
Psychiatrist (*n* = 33)	4.13 ± 0.67	2.62 ± 0.67	3.52 ± 0.75	2.92 ± 0.73	3.34 ± 0.57
Psychologist (*n* = 8)	4.10 ± 0.59	2.72 ± 0.75	3.55 ± 0.84	3.25 ± 0.68	3.43 ± 0.65
Nurse assistant (*n* = 15)	3.89 ± 0.77	3.00 ± 0.83	3.53 ± 0.57	3.31 ± 1.01	3.45 ± 0.50
Ward staff (*n* = 6)	3.43 ± 0.92	3.27 ± 0.77	3.70 ± 0.57	3.61 ± 0.49	3.50 ± 0.46
Residents in training (*n* = 21)	4.44 ± 0.35	2.98 ± 0.65	3.79 ± 0.78	3.20 ± 0.75	3.64 ± 0.52
	*p* = 0.006	*p* = 0.105	*p* = 0.783	*p* = 0.330	*p* = 0.355
Active Service					
MHC (*n* = 53)	4.32 ± 0.54	2.89 ± 0.72	3.82 ± 0.68	3.18 ± 0.78	3.59 ± 0.54
PH ward (*n* = 60)	3.96 ± 0.75	2.93 ± 0.68	3.51 ± 0.68	3.07 ± 0.83	3.41 ± 0.50
Others (*n* = 26)	4.29 ± 0.44	2.75 ± 0.60	3.50 ± 0.64	3.17 ± 0.60	3.46 ± 0.45
Currently unemployed MH (*n* = 17)	4.20 ± 0.49	2.99 ± 0.60	3.60 ± 0.67	3.23 ± 0.74	3.53 ± 0.47
	*p* = 0.013	*p* = 0.637	*p* = 0.079	*p* = 0.826	*p* = 0.278
Work experience	r= −0.18	r= −0.31	r= −0.19	r= −0.15	r= −0.29
(*n* = 156)	*p* = 0.024	*p* = 0.001	*p* = 0.015	*p* = 0.060	*p* = 0.001

*r*: Pearson’s correlation coefficient. *p*: statistical significance. MH: mental health. MHC: mental health center. HP: psychiatric hospitalization.

## Data Availability

The data presented in this study are available on request from the corresponding author.
